# The PerPAIN trial: a pilot randomized controlled trial of personalized treatment allocation for chronic musculoskeletal pain—a protocol

**DOI:** 10.1186/s40814-022-01199-6

**Published:** 2022-12-09

**Authors:** E. Beiner, D. Baumeister, D. Buhai, M. Löffler, A. Löffler, A. Schick, L. Ader, W. Eich, A. Sirazitdinov, C. Malone, M. Hopp, C. Ruckes, J. Hesser, U. Reininghaus, H. Flor, J. Tesarz, PerPAIN consortium

**Affiliations:** 1 Department of General Internal Medicine and Psychosomatics, Heidelberg University, Heidelberg, Germany; 2 Institute of Cognitive and Clinical Neuroscience, Central Institute of Mental Health, Medical Faculty Mannheim, Heidelberg University, Heidelberg, Germany; 3 Integrative Spinal Research Group, Department of Chiropractic Medicine, Balgrist University Hospital, University of Zürich, Zürich, Switzerland; 4 University of Zürich, Zürich, Switzerland; 5 Department of Public Mental Health; Central Institute of Mental Health, Medical Faculty Mannheim, Heidelberg University, Heidelberg, Germany; 6 Experimental Radiation Oncology, Medical Faculty Mannheim, Heidelberg University, Heidelberg, Germany; 7 Interdisciplinary Center for Clinical Trials, Johannes Gutenberg University Medical Center Mainz, Mainz, Germany

**Keywords:** Chronic pain, Treatment personalization, Precision therapy, Personalized allocation, Psychotherapy

## Abstract

**Background:**

The therapy of chronic musculoskeletal pain (CMSP) is complex and the treatment results are often insufficient despite numerous therapeutic options. While individual patients respond very well to specific interventions, other patients show no improvement. Personalized treatment assignment offers a promising approach to improve response rates; however, there are no validated cross-disease allocation algorithms available for the treatment of chronic pain in validated personalized pain interventions. This trial aims to test the feasibility and safety of a personalized pain psychotherapy allocation with three different treatment modules and estimate initial signals of efficacy and utility of such an approach compared to non-personalized allocation.

**Methods:**

This is a randomized, controlled assessor-blinded pilot trial with a multifactorial parallel arm design. CMSP patients (*n* = 105) will be randomly assigned 1:1 to personalized or non-personalized treatment based on a cluster assignment of the West Haven-Yale Multidimensional Pain Inventory (MPI). In the personalized assignment condition, patients with high levels of distress receive an emotional distress-tailored intervention, patients with pain-related interference receive an exposure/extinction-tailored treatment intervention and patients who adapt relatively well to the pain receive a low-level smartphone-based activity diary intervention. In the control arm, patients receive one of the two non-matching interventions. Effect sizes will be calculated for change in core pain outcome domains (pain intensity, physical and emotional functioning, stress experience, participant ratings of improvement and satisfaction) after intervention and at follow-up. Feasibility and safety outcomes will assess rates of recruitment, retention, adherence and adverse events. Additional data on neurobiological and psychological characteristics of the patients are collected to improve treatment allocation in future studies.

**Conclusion:**

Although the call for personalized treatment approaches is widely discussed, randomized controlled trials are lacking. As the personalization of treatment approaches is challenging, both allocation and intervention need to be dynamically coordinated. This study will test the feasibility and safety of a novel study design in order to provide a methodological framework for future multicentre RCTs for personalized pain psychotherapy.

**Trial registration:**

German Clinical Trials Register, DRKS00022792 (https://www.drks.de). Prospectively registered on 04/06/2021.

**Supplementary Information:**

The online version contains supplementary material available at 10.1186/s40814-022-01199-6.

## Introduction

### Background

As the leading symptom of a wide variety of musculoskeletal disorders, chronic pain is one of the most serious health problems worldwide [1] with enormous consequences on psychological as well as physical health [2, 3]. It is estimated that more than 20% of the world’s adult population suffer from chronic pain and every 10th person is diagnosed with chronic pain every year [4, 5]. While initial causes of pain can often be adequately treated (e.g. surgery, joint replacement, anti-inflammatory drugs/biologicals), in many cases, the pain persists and becomes chronic. This is especially true for chronic musculoskeletal pain (CMSP) conditions where a specific cause for the pain can no longer be detected [3, 5]. These complex persistent CMSP conditions are manifested by a kaleidoscope of symptoms that are temporally dynamic. Various therapies are available, and a multimodal treatment principle is generally recommended [5]. However, the treatment of CMSP is usually difficult and the treatment outcomes are unsatisfactory [4]. Various randomized controlled trials (RCTs) for the treatment of CMSP have led to negative results despite promising outcomes from preclinical and early clinical studies [4, 6, 7]. Accordingly, the most recent Cochrane review found that multimodal pain treatment achieves only small effects on pain and disability immediately after treatment, which had disappeared by follow-up [4].

There is variability between patients in their response to different pain therapies (even in effective treatments), which can significantly limit overall effects in clinical trials. Often psychobiological mechanisms of pain aetiology and maintenance are not considered [6, 8]. This led to calls for personalized pain therapy with a focus on specific disease-eliciting and disease-maintaining mechanisms and the development of empirically based algorithms that determine the optimal treatment or treatment combinations for individual patients to improve both the clinical care of patients with pain and the success rates of established pain management procedures [6, 9]. A major problem may be related to the fact that patients with comorbid mental disorders such as anxiety and depression and a high burden of psychobiological factors may be even more in need of specialized mechanism-based treatments tailored to their specific multiple needs [6, 10, 11]. Across individual diagnostic categories (e.g. fibromyalgia (FM), osteoarthritis (OA) or chronic non-specific back pain (CBP)), chronic pain may be maintained by similar psychobiological, including comorbid mechanisms leading to the need of cross-diagnostic treatment approaches taking into account relevant core mechanisms [6]. There are several core biological and psychological mechanisms that interact with CMSP in complex ways and can determine the development and maintenance and spread of comorbid psychopathology [12]. Variability of these biological and psychological mechanisms in different clinical presentations of chronic pain appears greater between individual patients than between different underlying diseases, suggesting that mechanistic etiologies of the pain chronicity process and subsequent successful treatment are likely to be at the individual level rather than at the disease level [13].

To implement a personalized treatment approach, however, the characteristics of individual patients or subgroups of patients that show common disease mechanism maintaining their pain, which increase or decrease the response to a particular treatment, need to be identified. Based on previous work, including our own, we can distinguish three subgroups of musculoskeletal pain patients: (1) patients who are characterized by extensive dysfunctional behaviours with high levels of fear of pain, proneness to the rewarding and punishing consequences of pain, (2) patients characterized by high levels of (interpersonal) distress and comorbid depression/anxiety and (3) a group that is not characterized by a specific psychobiological characteristics and comorbidity, commonly termed “adaptive copers” [14]. At this point in time, these mechanistic differentiations are solely based on cluster analyses of questionnaire data; however, we have identified pathomechanisms that may be specific for these subgroups [15, 16]. These include functional brain imaging results [15, 16], as well as evidence for specific mechanisms related to maladaptive aversive learning, memory processes, and reduced capacity for pain-memory extinction including fear of pain and anxiety [12, 17]. Similarly, we previously found that high levels of stress and emotional distress, exposure to psychological trauma and high levels of depression were associated with hyperalgesia to deep and widespread pain and that emotional abuse leads to exacerbated spinal pain summation in chronic pain patients [18].

Further, earlier evidence suggests that this clustering is associated with divergent treatment responses. In a feasibility study [19], we treated a group of CMSP patients with high level of emotional distress with an Emotional Distress Desensitization-tailored treatment approach (EDDT) using eye movement desensitization reprocessing (EMDR), focusing on alterations of distressing experiences [20]. We adapted the trauma EMDR treatment manual to the specific needs of CMSP patients with high level of emotional distress [21]. This emotional distress-tailored treatment induced a significant and clinically meaningful reduction of pain intensity and disability [19, 22]. Significantly, the therapy also led to a normalization of the aforementioned changes in the somatosensory function.

Based on findings on the role of maladaptive aversive learning, memory processes and reduced capacity for pain-memory extinction including fear of pain and anxiety involved in chronic pain, an operant treatment approach based on an increase in healthy behaviours and a decrease in pain behaviours was recently expanded to brain-based extinction retraining. This Pain Extinction and Retraining-tailored treatment approach (PERT) yielded excellent results in CMSP patients in pilot studies and increased effect sizes when specifically provided to patients characterized by high dysfunctionality, fear of pain and anxiety, whereas those with high levels of distress profited less [23].

In addition, we also developed and tested a new pain diary that focuses on pain-free phases and activity rather than pain and disability [17]. With this positive pain diary, we aim to investigate momentary mechanisms, prognostic markers and treatment outcomes in daily life using smartphone- and sensor-based ecological momentary assessment (EMA) [24]. We found significantly lower pain and stress and higher mood ratings at the end of a 4-week trial period when the positive activity diary was implemented [17]. Quality of life was significantly improved as well. Ecological momentary assessments provide unique opportunity for real-world, real-time, interactive, adaptive and personalized administration of interventions based on the dynamics of individuals’ experience and behaviour and their interaction with contextual factors in daily life [25, 26]. Therefore, implementing this new pain diary as an ecological momentary low-level diary intervention for real-time and real-world activity-based attention modulation (EMDI) may be a useful intervention for patients with pain but low levels of comorbidity. This EMDI therefore complements the other two approaches, which are more likely to address patients with high levels of emotional stress or dysfunctional behavioural patterns.

Although all three therapeutic approaches address different core mechanisms and patient subgroups, to our knowledge, there are no approaches to date that have investigated the improvement of efficacy through such precise therapy allocation. Furthermore, there is a lack of validated and cross-disease allocation algorithms that would allow a personalized allocation to the different pain interventions. Against this background, this trial will test the feasibility and safety of a personalized pain psychotherapy allocation with three different treatment modules and explore the initial signals of efficacy and utility of such an approach compared to non-personalized allocation.

### Objectives

The primary objective of this study is to investigate the feasibility and safety of a personalized pain psychotherapy approach with three different treatment modules—EDDT, PERT and EMDI. A secondary objective is the initial estimation of efficacy and utility of such a personalized approach compared to a non-personalized approach on different core pain outcome domains.

## Design

The overarching aim of our personalized pain psychotherapy allocation is to allocate an individual to a therapeutic intervention that fits the best based on the individual characteristics of the patient. In our study, we will focus on the allocation of patients to three well-established and well-validated pain patient clusters whose categorization is based on the widely used West Haven-Yale Multidimensional Pain Inventory (MPI) [27]. Based on this questionnaire, patients will be assigned to one of the three clusters [14]. According to their cluster’s assignment, patients will be allocated “personalized” to the best fitting psychological treatment. In the control arm of this study, patients will be allocated to a treatment that does not match their cluster assignment.

This study is part of the consortium ‘PerPAIN - Improving outcomes in chronic musculoskeletal pain through a personalized medicine approach’ funded by the German Federal Ministry of Research and Education (01EC1904A). This report focuses on the implementation of the pilot study subproject ‘Personalized treatment for chronic musculoskeletal pain: a randomized double-blind controlled trial with multifactorial parallel arm design’. The paper presents the study protocol for the trial, adhering to the Standard Protocol Items: Recommendations for Interventional Trials (SPIRIT) Statement [28], while the results of the trial will be reported in line with the CONSORT 2010 Statement: extension for randomized pilot and feasibility trials [29]. An additional file shows this in more detail (see Additional file 1). All participants must provide written informed consent before inclusion in the study. The study has been approved by the Ethics Research Committee II of the Faculty of Medicine, University of Heidelberg (2020-579N) and will be carried out in compliance with the Helsinki Declaration.

### Overview

**Fig. 1:**
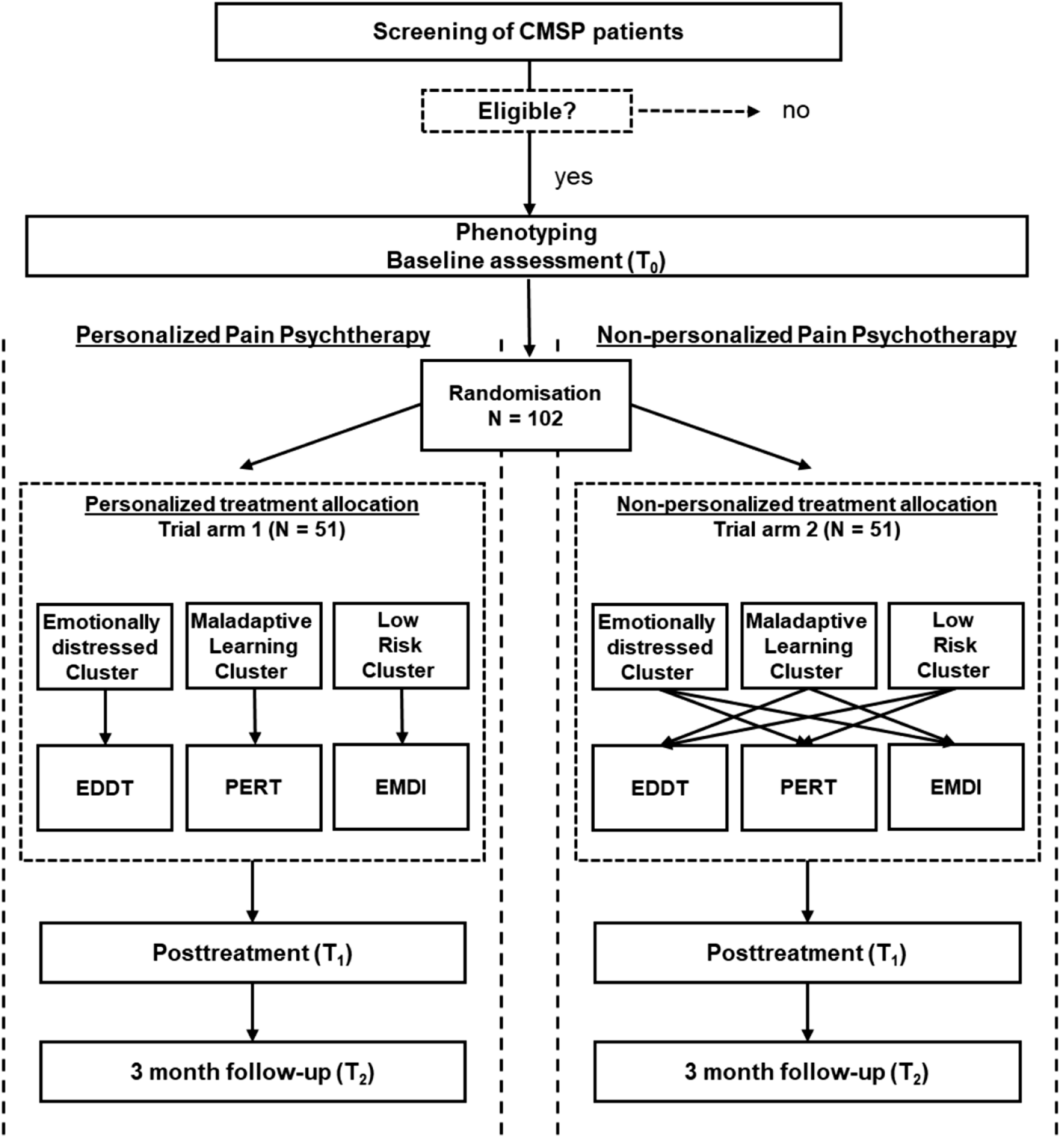
Intervention scheme/trial flow. Note: the same treatment interventions are applied in personalized as well as in non-personalized interventional study arms. However, the arms differ in the assignment to the respective treatment modules: While in the personalized assignment arms patients with high level of emotional distress receive an emotional distress-tailored intervention (EDDT), patients with high avoidance/maladaptive-learning behaviour receive an extinction/retraining-tailored treatment intervention (PERT) and patients with neutral risk profile receive a low-level diary intervention (EMDI), patients in the non-personalized arm receive a non-personalized intervention (i.e. patients with a high level of emotional distress receive either extinction/retraining-tailored or a low-level-tailored intervention, but not the emotional distress-tailored treatment). CMSP, chronic musculoskeletal pain; PERT, Pain Extinction and Retraining-Tailored Intervention; EDDT, Emotional distress desensitization and reprocessing-tailored intervention; EMDI, Ecological Momentary-tailored Low-Level Diary Intervention

Musculoskeletal pain patients will be offered participation in this study until at least 342 patients have been enrolled in baseline assessment and 105 patients have been included into our randomized controlled proof-of-concept study. All 105 patients enrolled in the study will undergo detailed phenotyping before and after intervention, which, together with the response/non-response data from the RCT, will serve to improve the allocation algorithm for future studies. All patients eligible will be randomly assigned 1:1 to a personalized or non-personalized treatment arm (Fig. 1). Personalization is achieved by a targeted allocation either to a therapy focused on emotional distress, a therapy focused on dysfunctional behaviours and maladaptive learning or allocation to a minimal intervention group where adaptive responses to pain will be registered. After baseline assessments (Fig. 1, T_0_) for relevant pathomechanisms, each patient included in the trial will receive either 12 weekly sessions of cluster-matching therapy (intervention arms)—where patients are allocated personalized (i.e. according to their individual pain mechanism)—or non-cluster-matching therapy (control arms) —where they are allocated non-personalized (i.e. randomly assigned between the other two interventions). After the last session, all procedures included in the baseline assessment will be performed as a posttreatment evaluation (Fig. 1, T_1_). Furthermore, a 3-month follow-up by means of telephone interview and questionnaire package will be implemented for patients taking part in the intervention study (Fig. 1, T_2_). At T_2_, the maintenance of the effects on pain and pain-related impairments will be assessed. In order to guarantee a correct evaluation of our primary outcomes in pain change, we will include an extended psychological assessment (details on outcomes are given below).

### Sample

The inclusion and exclusion criteria are chosen to ensure good representativeness and to cover the heterogeneity of different musculoskeletal pain conditions and diagnoses.

*Key inclusion criteria*: CMSP >3 months as symptom of (1) non-specific chronic back pain, (2) osteoarthritis, (3) fibromyalgia syndrome or (4) rheumatoid arthritis. Symptoms must be present for at least 3 months to ensure clinically relevant chronicity; the ability to see and use a mobile telephone (incl. with visual aids), age ≥18 years (no upper age limit) and ability to provide informed consent.

The *key exclusion criteria* will contain insufficient or unclear treatment of the underlying disease (according to the practitioner’s judgement), application for retirement/pension pending, on-going psychotherapy, severe, pharmaceutically treated acute life-threatening physical comorbidity or physical comorbidity which is incompatible with participation in the study according to the practitioner’s judgement, severe mental disorder (inability to consent, suicidality, psychosis spectrum disorders), neurological comorbidity (epilepsy, traumatic brain injury, seizures, multiple sclerosis, neurodegenerative diseases) and pregnancy. Participants will be excluded from the MRI assessment, if they have implants or metal parts in their body, which are not compatible with 3T MRI, or diseases which prevent lying still for 60 min. Clinical evaluation will be carried out by study physicians with experience in the diagnosis and management of chronic pain conditions. All patients will undergo a clinical evaluation by the Heidelberg Study Center for Clinical Pain Research within the Department of General Internal Medicine and Psychosomatics of Heidelberg University Hospital [30].

Participants of the CMSP group will be recruited through our tertiary pain clinic at the University Hospital Heidelberg, the affiliated academic teaching hospital of the University of Heidelberg in Baden-Baden as well as the Central Institute of Mental Health in Mannheim. Should the recruitment rate prove to be insufficient, recruitment will be extended to cooperating general and specialist medical practices in the Rhein-Neckar area. All prospective participants will be screened before inclusion in the study. Screening data will be confirmed by a study physician specifically trained in the assessment of chronic pain conditions. CMSP patients will be entered into the trial until the necessary sample size is reached.

During the planning phase of the study, we involved patient representatives of patients with CMSP. Further, members of relevant patient groups were invited to an initial focus group to ensure patient-friendly study procedures and to reduce the patient burden at all stages. In addition, all participants will be asked systematically about their experiences and impressions during the study and results will be discussed together with patient representatives at regular meetings. For the communication of the results to patients, we will organize a patient forum after the completion of the study.

### Treatment personalization

With our pilot study, we aim to prepare a multi-centre randomized controlled trial (planned RCT) to assess the feasibility and safety of a mechanism-based personalized assessment and treatment approach for CMSP patients across disorders. To further improve the personalized allocation algorithm for the planned RCT, a comprehensive characterization of the study participants will be performed with dynamic quantitative-sensory testing paradigms, immunological and neuroendocrine assessments, psychological questionnaire assessments, longitudinal data collection in everyday life (EMA, ECG and activity sensors) and neuroimaging (fMRI) pre- and post-treatment.

As this pilot study compares a personalized treatment allocation based on patient characteristics with a non-personalized treatment allocation, the same treatment interventions are applied in both study arms. However, arms differ in the assignment to the respective treatment modules. In the personalized assignment arm, patients with high level of emotional distress receive an emotional distress-tailored intervention (EDDT: Emotional Distress Desensitization-tailored treatment), patients with high avoidance/maladaptive-learning behaviour receive an exposure/extinction-tailored treatment intervention (PERT: Pain Extinction and Retraining-Tailored Intervention) and patients with neutral risk profile receive a low-level smartphone-based intervention (EMDI: Ecological Momentary-tailored Low-Level Diary Intervention). For every intervention, a special standard operation procedure (SOP) protocol is written regarding the 12-week design and special psychological/pain characteristics of the patient groups. Patients in the non-personalized arm receive a non-personalized intervention allocation (e.g. patients with a high level of emotional stress receive either exposure/extinction-tailored or a low-level smartphone-based intervention, but not the emotional stress-tailored treatment). For this study, matching to the subtypes will be based on a cluster analysis of the West Haven-Yale Multidimensional Pain Inventory (MPI) as well as the age and gender of participants, which have been identified as significant predictors of MPI clusters in previous research. Cluster status of individual participants will be identified by using soft k-means clustering of previous data collected from CMSP patients by the study teams. Using this data, clusters were identified, and the most likely cluster status for participants in the current study will be determined by an automated script implemented in an electronic data collection tool (REDCap) using a random-forest decision algorithm using the established clustering algorithm. Based on this algorithm, participants in the intervention arm will be assigned to the intervention that best fits according to their cluster, while patients in the control arm will be randomly assigned to one of the interventions that do not fit their cluster.

### Interventions

The individual interventions consist of 12 weekly face-to-face sessions (EDDT, PERT), or a minimal intervention condition in which smartphone-based diary queries are monitored daily over a period of 12 weeks (EMDI). The efficacy and safety for all three treatment modules have been demonstrated in previous studies, yet their superiority in outcomes depending on patient cluster has not been formally addressed. During the trial, patients are encouraged not to change their existing therapies over the course of the therapy. All additional treatments will be recorded regularly. In order to ensure that therapy protocols are appropriate and that therapists learn to follow these protocols in early stages of the study, a subsample of pilot participants will be invited to take part in the interventions without being part of evaluation of the personalized treatment allocation algorithm to aid the quality assurance of interventions.

#### Emotional distress desensitization and reprocessing (EDDT) intervention

EDDT is a stress-reducing intervention that combines the use of well-established trauma intervention elements (including imaginal exposure and cognitive and self-control techniques) and the use of specific EMDR elements such as bilateral sensory stimulation (e.g. left–right eye movements or bilateral hand-tapping induced by the therapist’s fingers) and the dual focus of attention principle. With the dual focus of attention principle, patients simultaneously focus on distressing memories and an external bilateral sensory stimulus. This procedure is suggested to facilitate information processing of emotionally distressing memories (e.g. traumatic events or pain sensations) and thereby cause a decreasing or even an elimination of the emotional distress related to these memories. Recent studies have shown that the dual application of bilateral sensory stimulation is highly efficient in triggering the inhibitory effects of the thalamus on the amygdala [31]. This strategy is increasingly being used to treat patients with chronic pain and its efficacy in pain treatments has been shown in several randomized controlled trials [22, 32]. Promising results have been reported especially for chronic musculoskeletal disorders, including back pain [33] and inflammatory joint pain [34]. The treatment protocol for this study is based on a standardized manual [21] and the possible targets for processing will encompass disturbing memories, current pain perceptions and pain-related fears and cognitions, and anticipated future painful situations together with the associated cognitions, emotions and bodily sensations. Participants who are allocated to the intervention group will receive a manualized and 12-session outpatient psychotherapeutic EDDT intervention (weekly session for 100min).

#### Pain Extinction and Retraining-tailored (PERT) intervention

PERT is a structured cognitive-behavioural derived intervention that focuses on retraining and alterations of maladaptive brain responses and includes elements such as activity increase, reduction of compensatory behaviours, medication management, altering social interactions, training of pleasant events and improving sensory discrimination. The treatment is based on an extension of a standardized manual [35] and consists of 12 weekly 100-min sessions led by two psychologists and is conducted in groups of 5–6 patients. To ensure a good patient-therapist relationship, all patients will have two individual sessions with the therapist prior to the group therapy. The group therapy includes video feedback of expressions of pain as well as training of pain-incompatible behaviours, increase of work-related and social activities and medication management. Patients are encouraged to participate in role plays to reduce dysfunctional pain behaviour and promote healthy behavioural responses. To increase the transfer of the treatment effects from the clinic to everyday life of the patients, the training also includes several sessions together with their spouses. Spouses will be fully informed and consented just as study participants. In previous studies, we could show that PERT interventions are superior to multimodal pain interventions and that maladaptive brain activation patterns can be altered by such interventions [15, 36–39]. In a study that is currently under review (Thieme, Kleinböhl et al., submitted), we analysed post hoc to what extent a matching of PERT to patients who are highly dysfunctional compared to those who are characterized by traumatic events/high stress/depressive responses or patients who have no specific dysfunctional or stress-related characteristics boost the treatment effect. Patients who happened to receive the fitting personalized treatment showed an almost 2-fold increase in effect size and a 50% reduction in drop-out. In this previous study, we did a post hoc analysis—the aim is now to a priori assign patients based on an optimized treatment algorithm.

#### Ecological momentary low-level diary intervention for activity-based attention modulation (EMDI)

We will use a positive activity diary as a low-level intervention with the goal of diverting the focus of attention away from pain and negative body and emotional experiences. This smartphone-based ecological momentary intervention app will not assess pain, negative mood or stress but the absence of pain, positive mood and positive events as targets for refocusing on positive events rather than pain. This will include enhancing, consolidating and interactive ecological momentary intervention components. In previous studies, we developed and tested a new pain dairy that focuses on pain-free phases and activity rather than pain and disability. We found significantly lower pain and stress and higher mood ratings at the end of a 4-week trial period when the positive activity diary was implemented (Nees et al., submitted). We therefore suggest that implementing this diary as an EMI may be a useful and safe low-level intervention for patients with pain who do not show any significant comorbidity or other psychobiological pathogenetic mechanisms.

### Randomization and allocation

For the allocation of the participants, a computer-generated list of random numbers will be used. Randomization for the pilot study will be carried out by the Interdisciplinary Center for Clinical Trials (IZKS) Mainz using permuted blocks of variable lengths. The randomization lists will be implemented in the electronic case report form system (eCRF) such that randomization can be performed electronically as new participants are added to the study. After providing informed consent and the acquisition of baseline data, participants will be randomly assigned to experimental intervention (personalized allocation) or control intervention (non-personalized allocation) via this eCRF system. Allocation will be managed by an independent study manager involved neither in sequence generation, assessments or treatment. All processes will be monitored and controlled for correctness by the IZKS Mainz. This process will be started only after the enrolled participants will have completed all baseline assessments and it is time to allocate the intervention. Randomization will be stratified by cluster, so that patients have a 50% chance of being allocated to the treatment matching their cluster, and 50% of being allocated to one of the two interventions (25% chance each) not matching their cluster.

### Blinding

Patients, therapists and the researchers carrying out participant assessments will be blinded to the respective condition (personalized vs. non-personalized), and success of blinding will be evaluated to explore possible source of bias. The double-blinding of patients and data collectors (i.e. patients, therapists and psychologists) is kept during the course of the study and statistical analysis will be also done with blinding maintained to avoid bias. Randomization authorities from the Interdisciplinary Center for Clinical Trials (IZKS) Mainz will be instructed to report any suspected breach of the blinding procedures.

### Sample size calculation

The study will be performed in an exploratory fashion and is planned as a pilot study to describe the feasibility and safety of personalized treatments for CMSP as a primary outcome, as well as an initial estimation of signals efficacy and utility as secondary outcomes. Even though a formal power calculation is not necessary in a pilot study, we nevertheless performed a power calculation to better estimate the initial signals of efficacy and utility.

For power considerations, we use a mixed-model with repeated measurements (MMRM), a two-sided significance level of 5% and a sample size of 90 patients (→45 per study arm →90 total from a total of 105 patients at an assumed dropout rate of 15%). The MMRM takes into account also the interim measurements for each patient and allows therefore a fuller use of the data than an ANCOVA. The sample size of 105 patients allows to calculate our feasibility measures with a 95% confidence interval width from 11 to 19% (for varying proportion 0.1–0.5) based on a Wald interval for binary measures [40]. Moreover, patients with data with at least one measurement can be used for the primary analysis; therefore, we expect fewer patients not to be usable for the analysis compared to an ANCOVA. Between repeated measurements, a correlation of 0.3 is assumed. Then, the study has a power of 80% to detect a true effect size of *f* = 0.22, a power of 85% for *f* = 0.23 and 90% power for a true effect size of *f* = 0.25. Dropouts will be analysed according to the underlying reasons for dropout and distinction will be made between those dropouts that are preventable by modification of study design and those that are not preventable by study design adaptations.

### Outcomes

The primary objective of this study is to investigate the feasibility and safety of a personalized pain psychotherapy approach with three different treatment modules. Based on the success of recruitment, randomization, assessment of outcomes, treatment adherence, treatment satisfaction, compliance and acceptability, clinical feasibility will be judged as ‘feasibility given’, ‘readjustment’ for the main trial necessary or ‘feasibility not given’.

Feasibility and safety outcomes will be documented and reported comparatively

1. *Recruitment*: number of participants recruited over the study period
  i. Successful recruitment of at least 105 participants (feasibility given)
  ii. Recruitment of 105 participants after modification of recruitment strategy (readjustment necessary)
  iii. Less than 105 participants after modification of recruitment strategy (feasibility not given)
2. *Assessment of inclusion criteria*: proportion of potential participants assessed after written consent obtained
  i. 95% of potential participants completed after giving written consent (feasibility given)
  ii. 75% of potential participants completed after giving written consent (readjustment necessary)
  iii. Less than 75% of participants completed after giving written consent (feasibility not given)
3. *Randomization*: number of participants successfully randomized after completion of eligibility screening and baseline assessments
  i. Successful randomization of at least 105 participants after completion of eligibility screening and baseline assessments (feasibility given)
  ii. Randomization of 90 participants (15% dropout rate) (readjustment necessary dependent of retention rate)
  iii. Randomization of less than 90 participants (feasibility not given)
4. *Retention* and *adherence*
  i. 85% retention rate of 105 randomized participants, for end-of-treatment assessment at least at one of the end-of-treatment assessments (T_1_/T_2_) (feasibility given)
  ii. Retention of less than 85% of randomized participants, but reasons for dropout can be prevented by modification of study design (readjustment necessary)
  iii. Retention rate of less than 85% of those dropouts that are preventable by modification of study design (feasibility not given)
5. *Treatment satisfaction, compliance and acceptability*
  i. Negative treatment effects: Unwanted and negative effects of the treatment will be assessed via the Negative Effects Questionnaire (NEQ) [41] supplemented by the subscales for negative effects on working place, partnership and family/peers of the Inventory for Assessing Negative Effects of Psychotherapy (INEP) [42].
  ii. Severe adverse events (SAEs) will be documented and reported descriptively.
  iii. Dropouts will be analysed according to the underlying reason for dropout and distinction will be made between those dropouts that are preventable by modification of study design and those that are not preventable by study design adjustment.

A secondary objective is the initial estimation of the efficacy and utility of such a personalized approach compared to a non-personalized approach on the different outcomes of the will outcome domains (pain intensity and physical functioning, emotional functioning and stress experience, participant ratings of improvement and satisfaction). Therefore, the exploratory analysis of the data will select the following candidate endpoints:

1. Pain severity: Pain severity will be assessed via the pain severity subscale of the Multidimensional Pain Inventory (MPI-D) [27]. This subscale summarizes the items pain now, pain in the last week and suffering related to pain. The MPI-D is a valid instrument with a high reliability (Cronbach’s α ≥ 0.90).
2. Disability/quality of life will be assessed via the Short-Form-Health Survey 12 (SF-12) and the Oswestry Disability Index (ODI). The SF-12 measures the impact of physical and mental health status on everyday life during the last 4 weeks. With the SF-12 the mental as well as the physical component can be assessed. It has a high validity and a good reliability for both, the mental and the physical component, with Cronbach’s *α* ≥ 0.75 and *α* ≥ 0.82, respectively [43]. The ODI captures the level of disability at the moment caused by pain in various activities of daily living (e.g. lifting weights, ability to care for oneself, ability to walk, ability to sit, sexual function, ability to stand, social life, quality of sleep and ability to travel). It is a validated questionnaire with good internal consistency [44].
3. Anxiety and depression: the level of anxiety and depression of the last week will be assessed via the Hospital Anxiety and Depression Scale (HADS). With 14 items, the HADS is a self-assessment scale that measures anxiety and depression through two subscales. Seven items for each subscale are rated by the patients on a 4-stage response format. It was in particular developed for somatic disorders and physical symptoms were therefore excluded. The HADS has high validity and reliability [45].
4. Positive and negative affect: Affectivity will be assessed via the Positive and Negative Affect Schedule (PANAS). This scale captures both positive and negative affectivity by asking for an assessment of positive and negative states of mind during the last week on a 5-point scale ranging from “not at all” to “extremely”, assessed on 20 items. It has a good validity and reliability with Cronbach’s α ≥ 0.86 [46, 47].
5. Global impression of change: Global impression of change will be assessed from both the patient’s (patient’s global impression of change, PGIC) and the therapist’s perspective (therapist’s global impression of change, TGIC). It is a 7-point scale with answers coded from “very much improved” to “very much worse”.
6. Days out of work (socioeconomic aspect) and change in medication since start of treatment will be assessed via additional items.
7. Activity levels will be assessed based on EMA sampling, with 10 queries per day, assessing for example pain intensity, attention to pain, pain catastrophizing, fear of pain and positive/negative affect.

For endpoints 1–6, the change between T_0_ and T_1_ (or 3-month follow-up) will be used as the outcome variable. In addition, the candidate endpoint domains will be investigated after 4 and 8 weeks to explore temporal therapy effects and dose-response relationships.

### Statistical analyses

Descriptive statistics (absolute and relative frequencies for variables with nominal and measures of position (mean, median) and variability measures (standard deviation, interquartile range and range) for variables with interval or ratio scaling) will be used to compare participant characteristics between the study arms.

Feasibility objectives will be quantified. If appropriate, 95% confidence intervals will be calculated based on the Wilson Score interval for binary measures. We favoured the Wilson Score interval against Wald interval due to their good properties for small numbers of trials, compared to Wei and Hutson [40]. For the analysis of the primary outcome, we will use the complete and pseudonymized data set and follow the intention-to-treat approach which includes all patients in the group they were allocated to by randomization. This approach preserves the allocation of treatment by randomization and it will be as close as possible to the ITT ideal of including all randomized patients. As sensitivity analysis, the analysis will be repeated in the per-protocol (PP) set, excluding patients with major protocol violations. Additional sensitivity analyses will explore the effects of the treatments the patients actually received. The null-hypothesis H0 (no difference occurs in the population means with regard to pain severity at T1 between the experimental intervention and the control intervention) will be tested against H1 (there is a difference in the population means of pain severity differences between the two groups at T1 and T0). Provided that the model assumptions are fulfilled, the null hypothesis will be tested using a (robust) mixed linear regression model at a significance level of 5%. The primary outcome (pain severity at T1, difference to T0) will be analysed using mixed-effects model repeated measurements after 4, 8 and 12 weeks (=T1). The analysis will be adjusted for condition, gender and baseline (T0) pain severity. Compound symmetry will be assumed. Because of the exploratory nature of the study, the observed initial signals of efficacy and utility will be considered more important than the *p*-values. The corresponding *p*-values of these tests will be interpreted purely descriptively. We will compute effect sizes and interpret them together with the respective 95% confidence intervals. Regarding secondary endpoints, exploratory data analysis will be performed using appropriate analytical methods depending on the respective parameter. Details of the statistical analyses will be fixed in a statistical analysis plan developed by the statistics department of IZKS Mainz prior to database closure. The safety analysis includes calculation and comparison of frequencies and rates of adverse events. Furthermore, statistical methods are used to assess the quality of data and the homogeneity of intervention groups. All analyses will consider gender aspects. Prior to all analyses, we will pre-specify a statistical analysis plan.

To more closely assess the ecological validity of our design in a naturalistic setting, a second analysis will be carried out where 1/3 of matched patients are randomly sampled and statistically reallocated to the non-matching arms. This is to allow a comparison between personalized treatment and truly random assignment (rather than matched vs non-matched), which may more closely mirror the ultimate implementation of personalization methods in clinical services or during a confirmatory multi-centre RCT.

### Feasibility

Feasibility will be assessed according to the following criteria: recruitment, assessment of inclusion criteria, randomization, retention and adherence, treatment satisfaction, compliance and acceptability. On the basis of these criteria, we will assess whether (1) feasibility is completely given, (2) feasibility is limited but likely to be achieved with adjustment of the study procedure or (3) feasibility is not given.

### Retention

We will continuously monitor the trial for any operational issues (i.e. failure in appointment management, no-show of patients). Concerning data collection, we will prioritize short questionnaires to reduce participant burden. To encourage retention at each study timepoint, non-responders will receive up to five reminders in total via phone, mail and e-mail. Outcome assessments may be completed in multiple sittings.

### Missing data

Applying the participant retention strategies outlined above, we will try to minimize the missing outcome data. Notwithstanding, we will record reasons participants are lost to follow-up. Prior to multiple imputation of missing values for primary and secondary outcomes at the item level, we will conduct sensitivity analyses to assess the robustness of the missing data assumption.

### Dissemination policy

Regardless of the magnitude or direction of effect, the results of this trial will be presented at relevant national and international conferences and as published articles in peer-reviewed journals. Publication of the study results will be based on the CONSORT-SPI 2018 statement for social and psychological interventions and the CONSORT extension for adverse effects. To reach health care policy and practice audiences (e.g. government bodies) concerning the scale-up of the model, we will present the findings at policy-maker- and service-provider-run conferences. Aiming at directly informing the work of policy-makers and practitioners, we will report the findings in plain language formats to them and compile an executive summary drawing together with key findings of all aspects of the intervention with a series of journal articles included as appendices.

### Trial status

At the time of submission, patient recruitment to the trial has commenced. The anticipated study completion date is November 2023**.** This trial was prospectively registered on the German Clinical Trials Register (DRKS) with study ID DRKS00022792 on April 6, 2021.

## Supplementary Information

**Additional file 1.** CONSORT 2010 statement: extension to randomized pilot and feasibility trials.

## Data Availability

Not applicable.

## Abbreviations

CBP: Chronic non-specific back pain
CMSP: Chronic musculoskeletal pain
EDDT: Emotional Distress Desensitization-tailored treatment approach
EMA: Ecological momentary assessment
EMDI: Ecological momentary low-level diary intervention
EMDR: Eye movement desensitization and reprocessing
FM: Fibromyalgia
HADS: Hospital Anxiety and Depression Scale
INEP: Inventory for Assessing Negative Effects of Psychotherapy
MMRM: Mixed-model with repeated measurements
MPI: West Haven-Yale Multidimensional Pain Inventory
NEQ: Negative Effects Questionnaire
OA: Osteoarthritis
ODI: Oswestry Disability Index
PANAS: Positive and Negative Affect Schedule
PERT: Pain Extinction and Retraining-tailored treatment approach
PGIC: Patient’s global impression of change
RCT: Randomized controlled trials
SAE: Severe adverse events
SF-12: Short-Form-Health Survey 12
